# Three‐year effectiveness and safety of the XEN gel stent as a solo procedure or in combination with phacoemulsification in open‐angle glaucoma: a multicentre study

**DOI:** 10.1111/aos.14886

**Published:** 2021-05-10

**Authors:** Herbert Reitsamer, Vanessa Vera, Simon Ruben, Leon Au, Jorge Vila‐Arteaga, Miguel Teus, Markus Lenzhofer, Andrew Shirlaw, Zhanying Bai, Mini Balaram, Ingeborg Stalmans

**Affiliations:** ^1^ Department of Ophthalmology and Optometry University Clinic Salzburg SALK/Paracelsus Medical University Salzburg Austria; ^2^ Department of Ophthalmology Unidad Oftalmologica de Caracas Caracas Venezuela; ^3^ Department of Ophthalmology Southend University Hospital NHS Foundation Trust Westcliff‐on‐Sea UK; ^4^ Department of Eye Research Manchester Royal Eye Hospital Manchester UK; ^5^ Medical Academic Health Science Centre University of Manchester Manchester UK; ^6^ Hospital Universitario La Fe Valencia Spain; ^7^ Hospital Universitario Principe de Asturias Universidad de Alcalá Madrid Spain; ^8^ Allergan, an AbbVie Company Marlow UK; ^9^ Allergan, an AbbVie Company Madison NJ USA; ^10^ Allergan, an AbbVie Company Irvine CA USA; ^11^ Department of Ophthalmology University Hospitals UZ Leuven Leuven Belgium

**Keywords:** glaucoma, intraocular pressure, gel stent, implant, MIGS, XEN

## Abstract

**Purpose:**

To assess the 3‐year effectiveness and safety of the XEN gel stent implanted ab interno in open‐angle glaucoma (OAG).

**Methods:**

This study was a multicentre, retrospective chart review of consecutive patients with OAG who underwent ab‐interno gel stent placement alone or combined with phacoemulsification between 1 January 2014 and 1 October 2015. Outcome measures included mean changes in intraocular pressure (IOP) and IOP‐lowering medication count from medicated baseline at 1, 2, 3 (primary outcome) and 4 years (if available) postimplantation. Intraoperative complications, adverse events of special interest (AESIs) and secondary surgical interventions (SSIs) were recorded.

**Results:**

The safety and effectiveness populations included 212 eyes (primary and secondary) and 174 eyes (primary), respectively. Mean IOP and medication decreased from 20.7 mmHg and 2.5 at baseline (*n* = 163 primary/first implanted eyes) to 13.9 mmHg and 1.1 medications (*n* = 76) at 3 years postimplantation, respectively. Mean changes from baseline in IOP (−5.6, −6.2 and −6.6 mmHg) and IOP‐lowering medication count (−1.8, −1.6 and −1.4) were statistically significant at 1, 2 and 3 years postimplantation, respectively. Results appeared comparable when implantation was performed with (*n* = 76) or without (*n* = 98) phacoemulsification. In primary eyes with 4‐year IOP and medication count data (*n* = 27), mean IOP was 14.0 mmHg on 1.3 medications at 4 years postimplantation. Fifteen (7.1%) eyes had intraoperative complications, 31 (14.6%) experienced 46 postoperative AESIs, and 26 (12.3%) required SSI.

**Conclusion:**

The gel stent effectively lowered IOP and IOP‐lowering medication count over 3 years, with a predictable and acceptable safety profile, when implanted via the traditional ab‐interno technique.

## Introduction

Glaucoma is the leading cause of irreversible blindness worldwide and is considered a global medical challenge (American Academy of Ophthalmology 2020; European Glaucoma Society 2020). Minimally invasive glaucoma surgery (MIGS) devices (Caprioli et al. 2015) are an important part of the glaucoma treatment armamentarium. The gel stent (XEN®45 Glaucoma Treatment System, Allergan, an AbbVie company, North Chicago, IL) is a less invasive surgical device that facilitates subconjunctival drainage similar to trabeculectomy. The stent is a 6‐mm‐long gelatin tube (Allergan 2016; Vera & Horvath 2014) with a 45‐µm lumen that connects the anterior chamber to the subconjunctival space.

In Europe, the gel stent is indicated for the reduction of intraocular pressure (IOP) in patients with primary open‐angle glaucoma (POAG) where previous medical treatments have failed (Allergan 2016). While previously published studies have shown effectiveness and safety of the gel stent at 2 years (single‐ and multicentre studies) (Gillmann et al. 2019; Reitsamer et al. 2019; Gabbay et al. 2020; Gillmann et al. 2020b) and 3 years (single‐centre study) (Gillmann et al. 2020a), this is the first multicentre study to report 3‐year effectiveness and safety of the 45‐µm gel stent in clinical settings.

## Methods

### Study design

This was a multicentre, non‐interventional, observational, retrospective chart review of medical records of patients with open‐angle glaucoma (OAG) who underwent gel stent implantation between 1 January 2014 and 1 October 2015 in 9 European centres (5 in Spain, 2 in the United Kingdom and 1 each in Austria and Belgium). The centres were identified based on response to a mailed qualification questionnaire that verified certification in the gel stent implantation and the availability of medical records from eyes that underwent placement of the gel stent during the proposed study period. All sites were trained in protocol and anonymized data entry requirements.

Before study initiation, the study protocol was approved by an independent ethics committee at each site, as required by local regulations in each country. The study was conducted in compliance with the tenets of the Declaration of Helsinki, European and national laws regarding data protection, and all local regulations applicable at the time of the study. The need for written patient consent was waived by all ethics committees in light of the retrospective study design, anonymized nature of the data collected and low risk of confidentiality breach, per the European General Data Protection Regulation.

### Patient identification and data extraction

Medical records from each site were screened to identify consecutive patients (1) who underwent placement of the gel stent as a standalone procedure (implant alone) or in combination with cataract surgery (phaco + implant) between 1 January 2014 and 1 October 2015; (2) who had a minimum required data set including age, gender, IOP measurements and number of IOP‐lowering medications used at baseline and during the study period; and (3) for whom the date of glaucoma‐related secondary surgical intervention (SSI), if any, was recorded. Patients not meeting all 3 criteria, and those who underwent gel stent implantation in combination with another procedure, were excluded. The targeted number of enrolled eyes was 150 (as detailed in the *Statistical analyses* section).

Each eye selected was assigned a unique identifier and data were extracted from the patient’s medical record, starting at the baseline visit through the date of the last follow‐up visit before 1 October 2018 (cut‐off date) or date of an SSI, whichever occurred first. Sites entered anonymized data into an electronic database chronologically, starting with eyes that underwent implantation closest to 1 January 2014.

Data collected included patient demographics, ocular and glaucoma‐related medical history and details of the surgical procedure (i.e. laterality, implant alone or phaco + implant, and use of antimetabolite and/or antifibrotic therapy), as well as IOP and the number of topical IOP‐lowering medications required at baseline and each follow‐up visit. Safety parameters that were collected included intraoperative and postoperative complications considered adverse events of special interest (AESIs), primarily based on the Directions for Use (Allergan 2016; Allergan 2017). Due to the retrospective nature of the study and anonymization of data, information on causality of AESIs was not collected. Also collected were best‐corrected visual acuity (BCVA), which was converted into LogMAR values for statistical analysis, visual field mean deviation (assessed per standard local practice and as available), as well as the details of other interventions performed on the study eyes, including needling and SSIs (e.g. trabeculectomy, second gel stent, trabecular micro‐bypass stent and trans‐scleral cycloablative procedures).

Patient visits of interest were categorized as baseline (day of the decision to implant the gel stent), the surgery day and postoperative follow‐up visits at months 12, 24, 36 and 48 (±3 months each), if data were available. Given that all eyes were required to have undergone gel stent implantation at least 3 years before cut‐off, it was anticipated that some eyes would have been followed for more than 3 years.

### Outcomes

The primary outcome measures were the mean changes in IOP and number of topical IOP‐lowering medications from medicated baseline, 3 years postimplantation. Secondary outcome measures included the mean changes in IOP and number of topical IOP‐lowering medications from medicated baseline at 1, 2 and 4 years postimplantation (if data were available at 4 years postimplantation), as well as the proportions of eyes achieving specific IOP reduction (≥20%, ≥30% and ≥40%) and IOP (≤18, ≤16 and ≤14 mmHg) levels. In addition, complete success, defined as ≥20% IOP reduction from medicated baseline without SSI, clinical hypotony (i.e. vision reduction of ≥2 lines related to macular changes consistent with hypotony maculopathy [macular folds], optic disc oedema and/or serious choroidal detachments because of IOP <6 mmHg) or topical IOP‐lowering medications, was evaluated at 1, 2, 3 and 4 years. Qualified success, defined as ≥20% IOP reduction from medicated baseline without SSI or clinical hypotony, while remaining on the same number or fewer topical IOP‐lowering medications, was also evaluated at 1, 2, 3 and 4 years postimplantation and did not include eyes that met the criteria for complete success. Overall success was defined as the sum of complete and qualified success at each time‐point. Any eyes requiring more IOP‐lowering medications at years 1 through 4 than at baseline would not qualify for success and were excluded from the success analyses.

Needling data were summarized based on the following time windows: ≤3 months, ≤6 months, ≤12 months, ≤24 months, ≤36 months and the entire study period.

### Statistical analyses

Two populations were specified for the analyses conducted. The safety population included all eyes of patients who met the eligibility criteria, both eyes being included in the analysis if implanted. The effectiveness population consisted of the first implanted eye (primary eye) of patients who met the eligibility criteria. Analysis of the primary outcome measures included patients who had both IOP and IOP‐lowering medication data at baseline (a subgroup of the effectiveness population). Sensitivity analyses (including primary and secondary eyes) were also conducted, as well as subgroup analyses based on the type of glaucoma (i.e. patients with POAG) or treatment received (i.e. implant alone versus phaco + implant).

Continuous variables were reported in terms of mean, standard deviation (SD), median and minimum and maximum, while categorical variables were reported in terms of number and percentage. Statistical analyses were carried out using SAS software version 9.4, and only observed data were reported, without imputation of missing data. All summarized data are presented in aggregate, and no site‐specific breakdown of data was performed to ensure that the data remained fully anonymized.

Based on data from the previously published APEX study (Reitsamer et al. 2019) (*N* = 199 patients) showing robust IOP control between months 1 (−6.3 mmHg) and 24 (−6.2 mmHg), a minimum IOP reduction from baseline of −5.6 mmHg (observed at month 3) was projected at 36 months in this study, with a 95% confidence interval (CI) of −7.2 to −4.0 mmHg. Considering a linear discontinuation rate of approximately 20% over 2 years in the APEX study (Reitsamer et al. 2019), the assumption that 30% of patients would not have data at 36 months in this real‐world study and the plan to accrue ≥60 eyes with ≥3 years of follow‐up data without SSI after gel stent implantation, the goal was to include 150 eyes in the study.

## Results

### Patient demographics, baseline characteristics and disposition

Data were collected from 174 consecutive patients (212 eyes with OAG); 38 (21.8%) patients had received the gel stent in both eyes. Given the predefined goal of including 150 eyes, screening was stopped once a total of 212 eyes were identified/reached, surpassing that goal. Overall, 52.3% of patients were female, 95.4% were White/Caucasian, and 73.6% were diagnosed with POAG (Table 1, 2). At baseline, mean (SD) age was 72.3 (10.8) years and mean (SD) IOP was 20.4 (5.1) mmHg on a mean (SD) of 2.4 (1.0) IOP‐lowering medications (Table 1). The number of eyes treated per site was as follows: 67, 31, 30, 24, 16, 15, 12, 9 and 8.

**Table 1:** Patient demographics and baseline characteristics (safety population).

Patient‐level data	Implant alone, *n* (%)	Phaco + implant, *n* (%)	Total, *n* (%)
	*N* = 98	*N* = 76	*N* = 174
Mean age (SD), y	69.4 (12.2)	76.0 (7.1)	72.3 (10.8)
Sex, *n* (%)			
Female	46 (46.9)	37 (48.7)	83 (47.7)
Male	52 (53.1)	39 (51.3)	91 (52.3)
Race, *n* (%)			
White	91 (92.9)	75 (98.7)	166 (95.4)
Asian	4 (4.1)	0	4 (2.3)
Other	3 (3.0)	1 (1.3)	4 (2.3)

Eye‐level data	*N* = 116	*N* = 96	*N* = 212
Study eye, *n* (%)			
Left	61 (52.6)	48 (50.0)	109 (51.4)
Right	44 (47.4)	48 (50.0)	103 (48.6)
Diagnosis, *n* (%)			
POAG	86 (74.1)	70 (72.9)	156 (73.6)
Pseudoexfoliation glaucoma	10 (8.6)	15 (15.6)	25 (11.8)
NTG	9 (7.8)	8 (8.3)	17 (8.0)
Pigmentary glaucoma	3 (2.6)	2 (2.1)	5 (2.4)
Uveitic glaucoma	0	1 (1.0)	1 (0.5)
Other	8 (6.9)	0	8 (3.8)
Mean IOP (SD), mmHg^a^	20.4 (4.7)	20.4 (5.5)	20.4 (5.1)
Mean number of IOP‐lowering medications (SD)^b^	2.6 (1.0)	2.3 (1.1)	2.4 (1.0)
Median	3.0	2.0	3.0
Min, max^c^	0, 4	0, 4	0, 4

IOP = intraocular pressure, max = maximum, min = minimum, NTG = normal‐tension glaucoma, phaco = phacoemulsification with intraocular lens placement, POAG = primary open‐angle glaucoma, SD = standard deviation.

^a^ Baseline data were similar in the effectiveness population (*n* = 174 primary eyes), with mean (SD) IOPs of 21.0 (4.6) mmHg (implant alone), 20.2 (5.6) mmHg (phaco + implant) and 20.7 (5.1) mmHg (total).

^b^ Baseline data were similar in the effectiveness population with both IOP and IOP‐lowering medication data at baseline (*n* = 163/174 eyes); the mean (SD) and median numbers of topical IOP‐lowering medications were 2.6 (1.0) and 3.0 (implant alone), 2.4 (1.0) and 2.0 (phaco + implant), and 2.5 (1.0) and 3.0 (total).

^c^ Five eyes that were not receiving any topical IOP‐lowering medications at baseline were implanted with the gel stent; the reasons for implanting were intolerance/allergy to topical IOP‐lowering medications (*n* = 2), not available (*n* = 2) and other (*n* = 1). All treatment‐related decisions were based on the investigators’ experience and judgement. Of those 5 eyes, 2 (1 in each treatment groups) were included in the primary effectiveness population.

The safety population included all 212 eyes, which had received the gel stent at least 3 years before the study cut‐off date and, therefore, could contribute data to the primary outcomes analyses if data were available/recorded. Of the 212 eyes, 174 eyes with IOP data at baseline were deemed to be primary eyes (effectiveness population) and, of those, 163 eyes had both IOP measurement and the number of topical IOP‐lowering medications recorded at baseline. Gel stent placement was performed in combination with phacoemulsification in 96 (45.3%) of the 212 eyes in the safety population and in 76 (43.7%) of the 174 eyes in the effectiveness population (Table 2). At years 1, 2 and 3, 151/174, 117/174 and 85/174 eyes contributed to the effectiveness measures, respectively.

**Table 2:** Eye disposition.

Safety population			
Visit (years)	Implant alone, *n* (%) *N* = 116	Phaco + implant, *n* (%) *N* = 96	Total, *n* (%) *N* = 212
1	100 (86.2)	82 (85.4)	182 (85.8)
2	80 (69.0)	61 (63.5)	141 (66.5)
3	59 (50.9)	42 (43.8)	101 (47.6)
4	16 (13.8)	17 (17.7)	33 (15.6)

Effectiveness population			
Visit (years)	Implant alone, *n* (%) *N* = 98	Phaco + implant, *n* (%) *N* = 76	Total, *n* (%) *N* = 174^a^
1	85 (86.7)	66 (86.8)	151 (86.8)
2	67 (68.4)	50 (65.8)	117 (67.2)
3	52 (53.1)	33 (43.4)	85 (48.9)
4	15 (15.3)	15 (19.7)	30 (17.2)

The numbers and corresponding percentages at 1, 2, 3 and 4 years refer to eyes that completed those indicated visits.

IOP = intraocular pressure, phaco = phacoemulsification with intraocular lens placement.

^a^ This analysis included all eyes that had IOP data at baseline. Analysis of the primary outcome measures included patients who had both IOP and IOP‐lowering medication data at baseline (a subgroup [*n* = 163] of the effectiveness population).

Thirty‐five (16.5%) of the 212 eyes (31/174 patients) were eligible to contribute 4‐year follow‐up data (Table 2). Of those, 33 and 30 were included in the 4‐year safety and effectiveness analyses, respectively.

### Effectiveness at 3 years

**Fig. 1:**
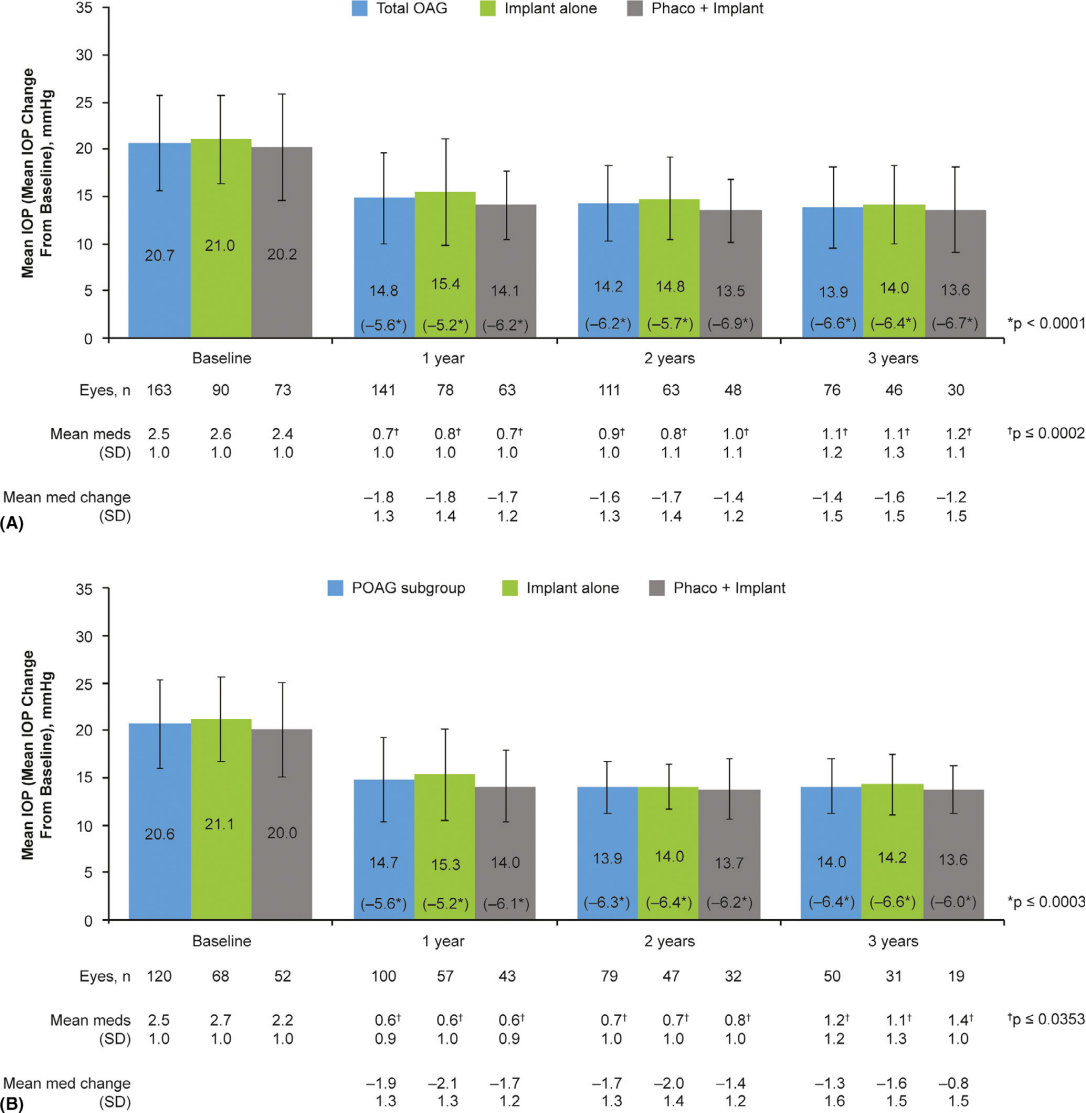
Mean and changes in mean IOP and number of IOP‐lowering medications from baseline over time in the total effectiveness.^a^ OAG population with both IOP measurement and number of topical IOP‐lowering medications recorded at baseline (*n* = 163^b^) (A) and patient subgroup with POAG (*n* = 120) (B). Mean IOP data are shown with standard deviations. p values were based on paired *t*‐tests evaluating the difference in IOP or number of topical IOP‐lowering medications from baseline to the indicated postoperative visit; p < 0.05 was considered statistically significant. ^a^All effectiveness analyses included one eye per patient; in cases of bilateral implantation, only the first implanted eye was analysed. ^b^11 patients were excluded from analysis of the primary outcome measures as they did not have both IOP and IOP‐lowering medication count data at baseline. IOP = intraocular pressure, meds = medications, phaco = phacoemulsification with intraocular lens replacement, OAG = open‐angle glaucoma, POAG = primary open‐angle glaucoma, SD = standard deviation.

At 3 years postimplantation in the effectiveness population that had both IOP measurement and the number of topical IOP‐lowering medications recorded at baseline (*n* = 163), the mean (SD) IOP decreased from 20.7 (5.1) mmHg on 2.5 (1.0) medications at baseline to 13.9 (4.3) mmHg on 1.1 (1.2) medications (primary outcome measures; *n* = 76 eyes). The mean changes from baseline in IOP (−5.6, −6.2 and −6.6 mmHg) and IOP‐lowering medication count (−1.8, −1.6 and −1.4) were statistically significant at 1, 2, and 3 years postimplantation, respectively (Fig. 1A). Similar results were found in sensitivity analyses (p < 0.0001), in which both primary and secondary eyes were included (*n* = 171, 135 and 92 at 1, 2 and 3 years, respectively), and in the corresponding treatment subgroups (implant alone, p < 0.0001; phaco + implant, p ≤ 0.0002) at all postbaseline visits.

**Fig. 2:**
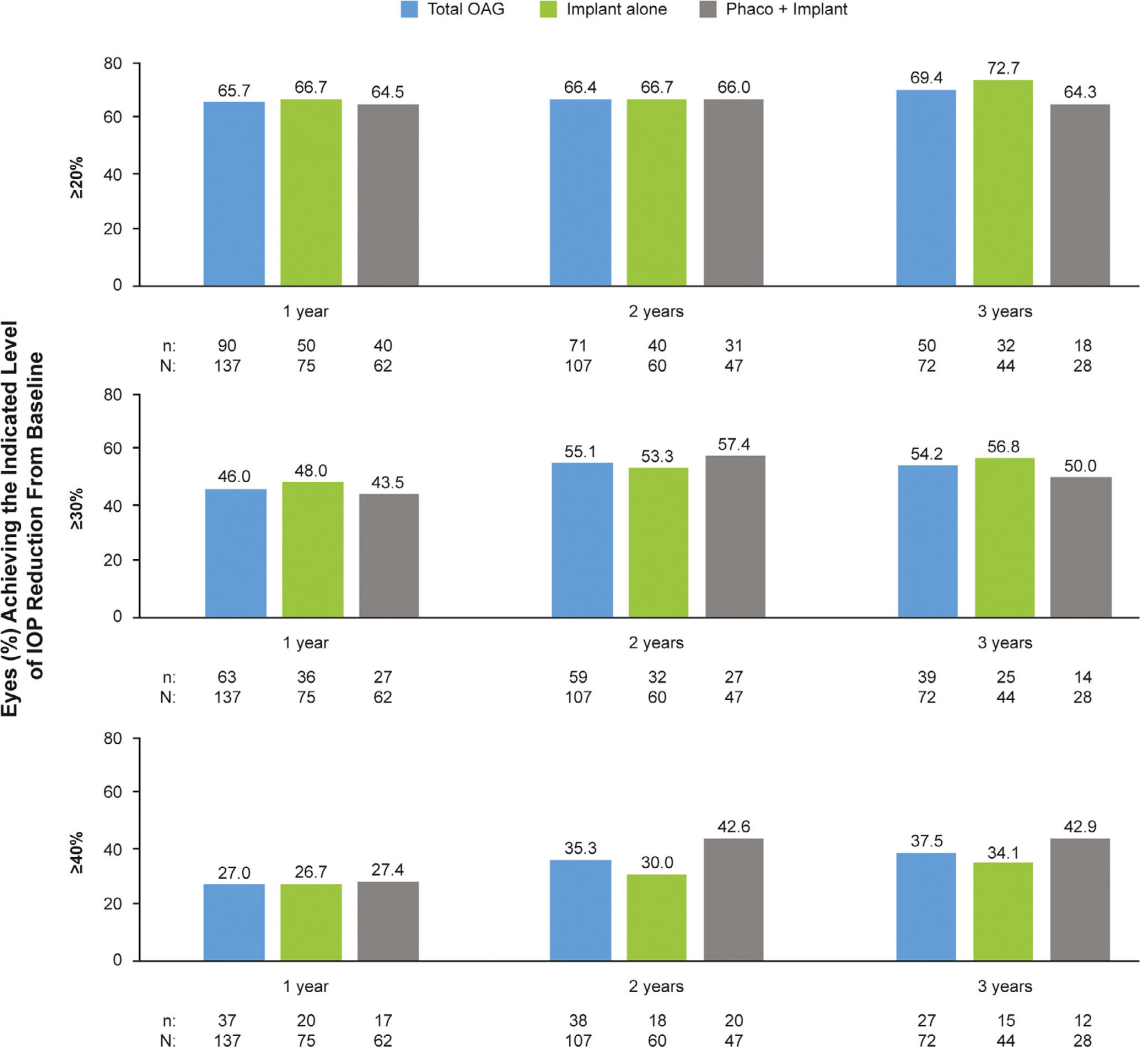
Proportion of eyes achieving ≥20%, ≥30% and ≥40% IOP reduction from baseline at 1, 2 and 3 years postimplantation of the gel stent in the effectiveness^a^ OAG population (*n* = 174^b^). ^a^All effectiveness analyses included one eye per patient; in cases of bilateral implantation, only the first implanted eye was analysed. ^b^This analysis included all patients who had IOP data at baseline. IOP = intraocular pressure, OAG = open‐angle glaucoma, phaco = phacoemulsification with intraocular lens placement.

**Fig. 3:**
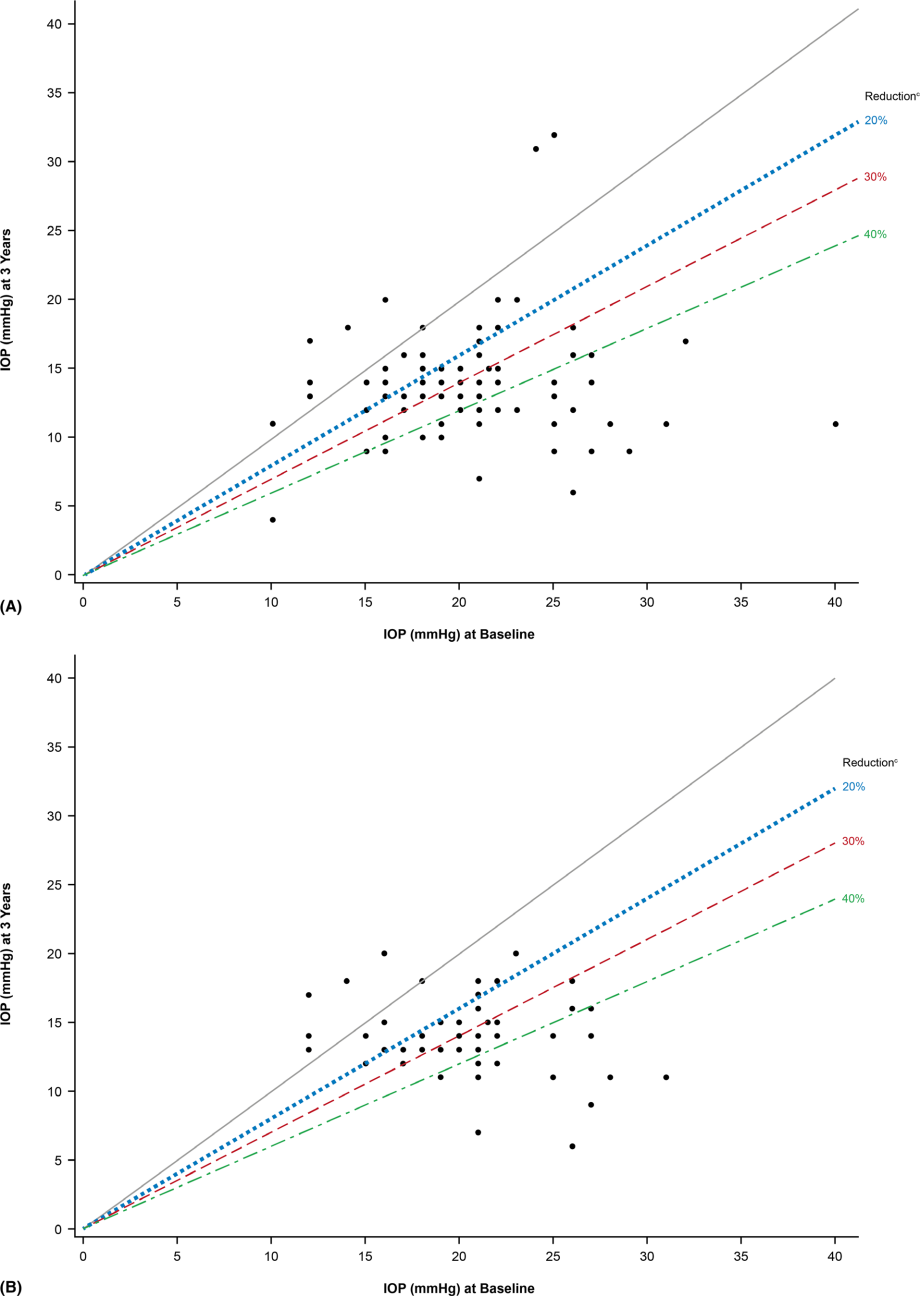
Scatter plots of IOP reduction as a function of preoperative IOP at 3 years in the effectiveness^a^ OAG population (*n* = 174^b^) (A) and patient subgroup with POAG (*n* = 123) (B). Each data point represents one eye. The grey line delineates IOP reduction (lower portion) from IOP increase (upper portion), relative to baseline IOP. Data points below the 20%, 30% and 40% IOP reduction lines achieved that level of IOP lowering or more. ^a^All effectiveness analyses included one eye per patient; in cases of bilateral implantation, only the first implanted eye was analysed. ^b^This analysis included all patients who had IOP data at baseline. ^c^Indicates IOP reduction success, as defined in the protocol. IOP = intraocular pressure, OAG = open‐angle glaucoma; POAG = primary open‐angle glaucoma.

**Fig. 4:**
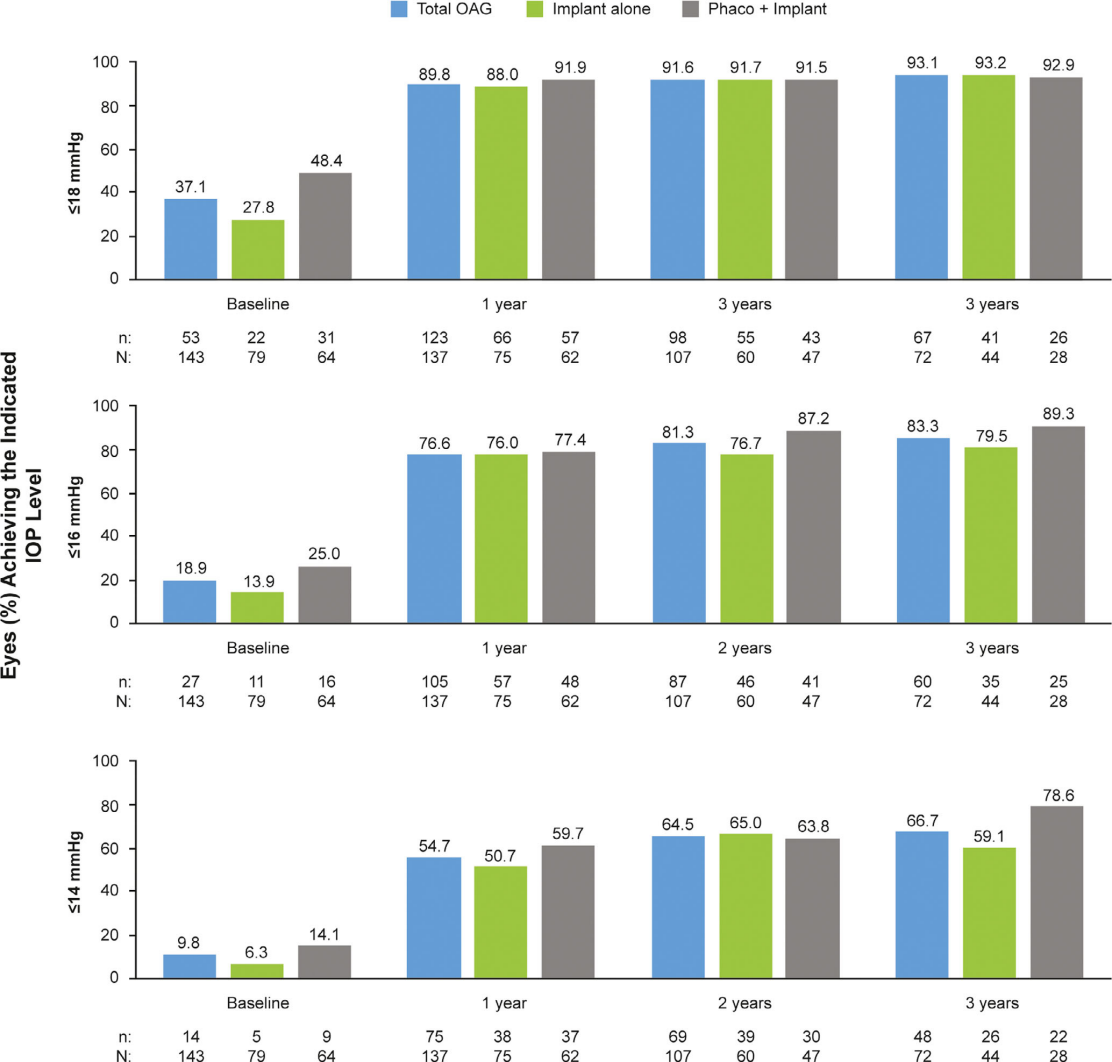
Proportion of eyes achieving IOP ≤18, ≤16 and ≤14 mmHg at 1, 2 and 3 years postimplantation of the gel stent in the effectiveness^a^ OAG population (*n* = 174^b^). ^a^All effectiveness analyses included one eye per patient; in cases of bilateral implantation, only the first implanted eye was analysed. ^b^This analysis included all patients who had IOP data at baseline. IOP = intraocular pressure, OAG = open‐angle glaucoma, phaco = phacoemulsification with intraocular lens placement.

**Fig. 5:**
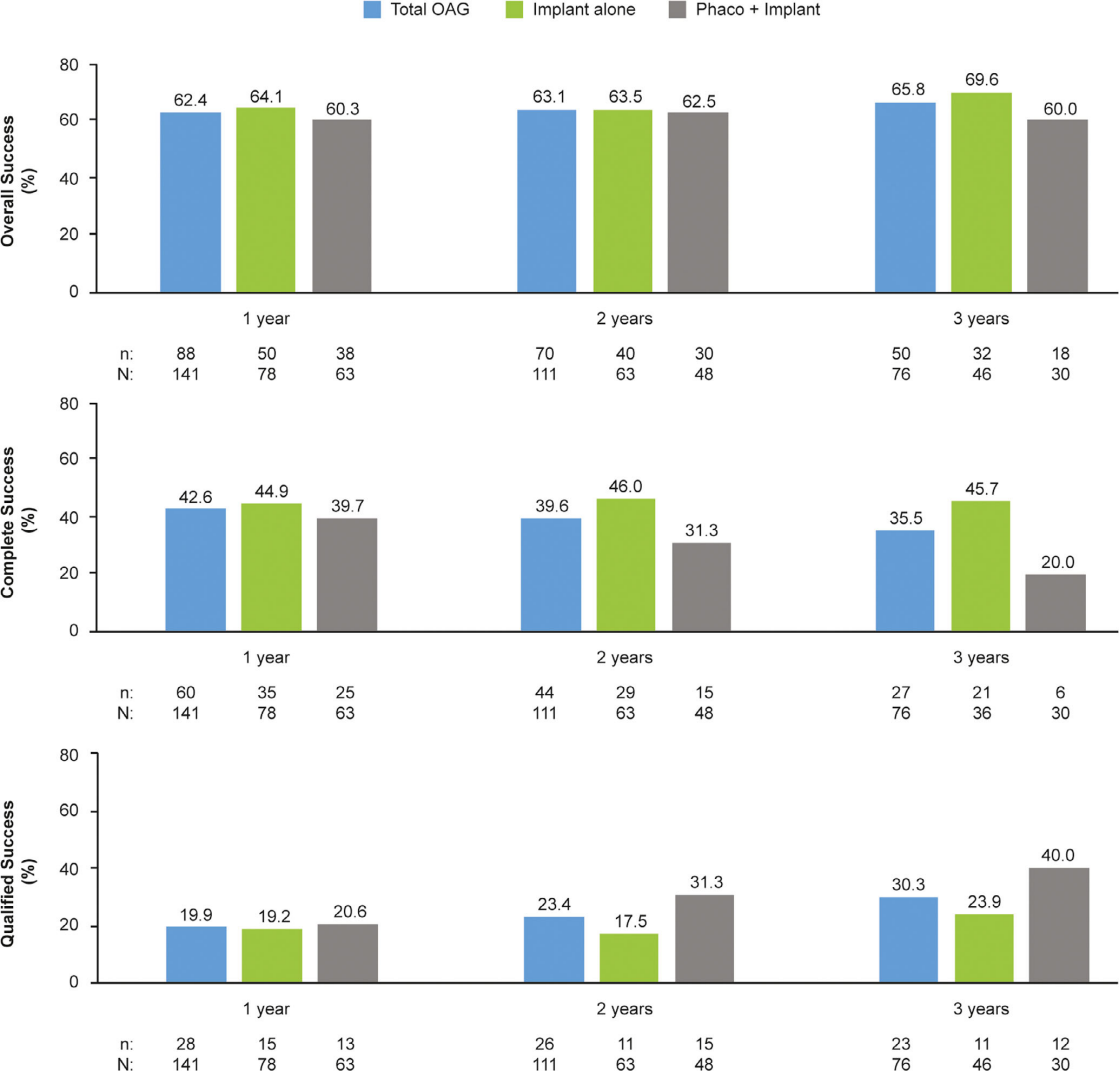
Rates of overall, complete and qualified success at 1, 2 and 3 years postimplantation of the gel stent in the effectiveness OAG population (*n* = 174)^a,b^. ^a^All effectiveness analyses included one eye per patient; in cases of bilateral implantation, only the first implanted eye was analysed. ^b^This analysis included all patients who had IOP data at baseline. Overall success was defined as the sum of complete success and qualified success. Complete success was defined as ≥20% IOP reduction from medicated baseline without SSI, clinical hypotony (as defined in the *Outcomes* section) or topical IOP‐lowering medications, analysed in the effectiveness population. Qualified success was defined as ≥20% IOP reduction from medicated baseline without SSI or clinical hypotony while remaining on the same number or fewer topical IOP‐lowering medications, analysed in the effectiveness population, and did not include eyes that met the criteria for complete success. Eyes that required more topical IOP‐lowering medications at years 1 (implant, *n* = 3; phaco + implant, *n* = 1), 2 (implant, *n* = 3; phaco + implant, *n* = 1) and 3 (implant, *n* = 2; phaco + implant, *n* = 2), compared with baseline, were excluded from the success analyses. IOP = intraocular pressure, OAG = open‐angle glaucoma, phaco = phacoemulsification with intraocular lens placement, SSI = glaucoma‐related secondary surgical intervention.

The proportions of eyes in the effectiveness population that achieved ≥20%, ≥30% and ≥40% IOP reduction from baseline were 69.4%, 54.2% and 37.5% at 3 years, respectively (Fig. 2); a scatter plot of the IOP reduction as a function of preoperative IOP at 3 years is presented in Fig. 3A. It is also notable that the proportions of eyes in the effectiveness population with IOP ≤18, ≤16 and ≤14 mmHg were at least 2.4, 4.1 and 5.6 times higher at years 1, 2 and 3, respectively, than at baseline (Fig. 4). In line with these findings, the rate of overall success was remarkably stable from year 1 through year 3 in the effectiveness population (62%–66%) (Fig. 5). Complete success and qualified success (as defined in the *Outcomes* section) were achieved in 35.5% and 30.3% of eyes at 3 years postimplantation, respectively (Fig. 5). Four eyes required more topical IOP‐lowering medications at years 1 (implant, *n* = 3; phaco + implant, *n* = 1), 2 (implant, *n* = 3; phaco + implant, *n* = 1) and 3 (implant, *n* = 2; phaco + implant, *n* = 2), compared with baseline, and were excluded from the success analyses.

### Effectiveness at 4 years

In total, 35 eyes underwent gel stent placement 4 years before the cut‐off date and, therefore, could have contributed to the 4‐year outcomes analysis (Table 2). Of those, 27 were primary eyes (defined in the *Statistical analyses* section) included in the effectiveness population and had a baseline IOP measurement and the number of topical IOP‐lowering medications recorded. Mean changes from baseline in IOP and number of IOP‐lowering medications in those 27 eyes were statistically significant at 4 years postimplantation (Table 3). Mean (SD) change in IOP from baseline (*n* = 27 eyes) was 5.4 (5.1) mmHg on 1.3 (1.3) medications at 4 years postimplantation.

**Table 3:** Effectiveness data at 4 years postimplantation of the gel stent

48‐month visit	Implant alone *N* = 16 eyes	Phaco + implant *N* = 15 eyes	Total *N* = 31 eyes^a^
Mean (SD) IOP change from medicated baseline, mmHg	−5.5 (5.0)	−5.3 (5.3)	−5.4 (5.1)
*n*	12	15	27
p value (compared with baseline)	0.0030	0.0018	<0.0001
Mean (SD) change in topical IOP‐lowering medication count from baseline	−1.1 (1.1)	−0.8 (1.4)	−0.9 (1.3)
*n*	12	15	27
p value (compared with baseline)	0.0053	0.0472	0.0008
Eyes achieving the indicated level of IOP reduction from baseline, *n*/*N* (%)^b^			
≥20%	7/11 (63.6)	8/13 (61.5)	15/24 (62.5)
≥30%	5/11 (45.5)	5/13 (38.5)	10/24 (41.7)
≥40%	3/11 (27.3)	4/13 (30.8)	7/24 (29.2)
Eyes achieving the indicated IOP level, *n*/*N* (%)^b^			
≤18 mmHg	9/11 (81.8)	13/13 (100)	22/24 (91.7)
≤16 mmHg	7/11 (63.6)	12/13 (92.3)	19/24 (79.2)
≤14 mmHg	5/11 (45.5)	8/13 (61.5)	13/24 (54.2)
Overall success rates, *n*/*N* (%)^c^	7/12 (58.3)	8/15 (53.3)	15/27 (55.6)

p values were based on paired *t*‐tests evaluating the difference in IOP or number of topical IOP‐lowering medications from baseline to the indicated postoperative visit; p < 0.05 was considered statistically significant.

IOP = intraocular pressure, phaco = phacoemulsification with intraocular lens placement, SD = standard deviation, SSI = glaucoma‐related secondary surgical intervention.

^a^ Eyes in the effectiveness population that had both an IOP measurement and number of topical IOP‐lowering agents. All effectiveness analyses included one eye per patient; in cases of bilateral implantation, only the first implanted eye was analysed.

^b^ Included in these analyses were patients who stayed on the same number or fewer topical IOP‐lowering medications and did not have clinical hypotony (as defined in the *Outcomes* section).

^c^ Overall success was defined as the sum of complete success and qualified success. Complete success was defined as ≥20% IOP reduction from medicated baseline without SSI, clinical hypotony or topical IOP‐lowering medications, analysed in the effectiveness population. Qualified success was defined as ≥20% IOP reduction from medicated baseline without SSI or clinical hypotony while remaining on the same number or fewer topical IOP‐lowering medications, analysed in the effectiveness population, and did not include eyes that met the criteria for complete success.

The proportions of eyes that achieved ≥20%, ≥30% and ≥40% IOP reduction from baseline postimplantation were consistent with those observed at years 1 to 3 (Table 3; Fig. 2). Likewise, the proportions of eyes with IOP ≤18, ≤16 and ≤14 mmHg at 4 years postimplantation were in line with those seen at years 1 to 3 (Table 3; Fig. 4). Outcomes at 4 years, including success rates, appeared to be consistent with those eyes followed for up to 3 years (Table 3; Fig. 5). Three eyes required more topical IOP‐lowering medications at year 4 (implant, *n* = 1; phaco + implant, *n* = 2) than baseline and were excluded from the success analyses.

### Effectiveness − POAG subgroup

In primary eyes with a diagnosis of POAG (*n* = 123, effectiveness population), the mean (SD) IOP decreased from 20.6 (4.7) mmHg on 2.5 (1.0) medications at baseline to 14.0 (2.9) on 1.2 (1.2) medications at 3 years postimplantation. The mean changes from baseline IOP (−5.6, −6.3 and −6.4 mmHg) and IOP‐lowering medication count (−1.9, −1.7 and −1.3) were statistically significant at 1, 2 and 3 years postimplantation, respectively (p < 0.0001 for all; Fig. 1B). In this POAG subgroup, statistically significant reductions in mean IOP (p ≤ 0.0003) and mean number of IOP‐lowering medications (p ≤ 0.0353) from baseline were observed at 1, 2 and 3 years whether implantation was performed as a solo procedure or in combination with phacoemulsification (Fig. 1B).

In an evaluation of specific IOP targets achieved in the subgroup of eyes with POAG, the percentage of eyes with available data that achieved IOP reductions ≥30% at 3 years postimplantation (*n* = 24/47, 51.1%) was similar to that reported in all eyes of the effectiveness population (*n* = 39/72, 54.2%) (Fig. 3). Also similar to findings reported at 3 years postimplantation in all eyes of the effectiveness population (Fig. 5) were the rates of overall (68.0%), complete (34.0%) and qualified (34.0%) success in the POAG subgroup at 3 years postimplantation.

### Effectiveness − Implant alone and phaco + implant subgroups

Although no formal statistical comparison of these treatment subgroups was performed, the gel stent effectively reduced IOP and the number of topical hypotensive medications at years 1, 2 and 3, whether eyes received the implant alone or in combination with cataract surgery (Fig. 1). The proportions of eyes that achieved ≥20%, ≥30% and ≥40% IOP reduction from baseline and those achieving target IOP levels ≤18, ≤16 and ≤14 mmHg at 1, 2 and 3 years postimplantation generally appeared comparable between treatment subgroups (Figs. 2, 4). Moreover, the rate of overall success was stable from year 1 through year 3 in both the implant alone (64%–70%) and phaco + implant (60%–63%) subgroups (Fig. 5). In those subgroups, complete success at 3 years was 45.7% and 20.0%, while qualified success was 23.9% and 40.0%, respectively, suggesting that despite seemingly comparable results overall between the implant alone and phaco + implant subgroups, the latter subgroup appears to be receiving more medications.

### Antimetabolite/antifibrotic use during implantation of the gel stent

Use of an antimetabolite/antifibrotic agent during the implantation procedure was documented in 203 (95.8%) of the 212 eyes in the safety population. Mitomycin C (MMC) was used in 198/203 (97.5%) eyes; an injection of steroid or anti‐vascular endothelial growth factor (VEGF) was used in 19 (9.4%) and 22 (10.8%) eyes, respectively, with some eyes receiving more than one agent. These agents were administered via subconjunctival injection (*n* = 193/239 – MMC, steroid and anti‐VEGF) or sponge application following conjunctival dissection (*n* = 7/239 – MMC, 0.2 mg/ml); in some cases, the route of administration was unknown (*n* = 8/239 eyes) or unavailable (*n* = 31/239 eyes).

The following absolute doses were documented for MMC injections: 5 µg (3.5%), 10 µg (47.0%), 20 µg (26.7%) and 40 µg (0.5%); the dose was not documented for 34 eyes.

### Needling

The number (%) of eyes that required needling by years 1, 2 and 3 postimplantation was 78 (37%), 89 (42%) and 91 (43%), respectively, and the mean number of needlings per eye was 1.3. Of the 212 eyes implanted, 119 (56%) required no needling during the study period, whereas 54 (26%), 20 (9%) and 19 (9%) required 1, 2 and 3 needlings, respectively. No eye received more than 3 needlings. The total number of needlings performed during the study period was 167; the majority (115, 68.9%) were performed within 12 months of implantation, with 46 (27.5%) taking place in the first 3 months. It is also worth noting that the majority of needling procedures performed during the first, second and third year postimplantation involved antimetabolite/antifibrotic agents (Table 4).

**Table 4:** Needling procedure performed during the study period and related variables.^a^

	≤1 year	≤2 years^b^	≤3 years^b^
All eyes, *n* (%)	78 (36.8)	89 (42.0)	91 (42.9)
Total needling procedures, *n*	115	145	163
Antimetabolite/antifibrotic use, *n* (%)			
Yes	92 (80.0)	117 (80.7)	134 (82.2)
No	23 (20.0)	28 (19.3)	29 (17.8)
Mean (SD) IOP, mmHg			
Preneedling	22.0 (7.5)	22.2 (7.5)	22.0 (7.4)
Postneedling	15.4 (6.3)	16.0 (6.8)	16.4 (7.1)
Overall success rate, *n*/*N* (%)^c^	38/103 (36.9)	34/81 (42.0)	29/60 (48.3)

IOP = intraocular pressure, SD = standard deviation, SSI = glaucoma‐related secondary surgical intervention.

^a^ Safety population unless otherwise noted.

^b^ This analysis is cumulative and includes needlings performed up to months 24 and 36 of the study, respectively.

^c^ Overall success was defined as the sum of complete success and qualified success. Complete success was defined as ≥20% IOP reduction from medicated baseline without SSI, clinical hypotony (as defined in the *Outcomes* section) or topical IOP‐lowering medications, analysed in the effectiveness population. Qualified success was defined as ≥20% IOP reduction from medicated baseline without SSI or clinical hypotony while remaining on the same number or fewer topical IOP‐lowering medications, analysed in the effectiveness population, and did not include eyes that met the criteria for complete success.

Among needled patients with available data at baseline and year 1, baseline and year 2, and baseline and year 3, mean (SD) IOP decreased from 22.0 (7.5), 22.2 (7.5) and 22.0 (7.4) mmHg preneedling to 15.4 (6.3), 16.0 (6.8) and 16.4 (7.1) mmHg postneedling at years 1, 2 and 3, respectively (Table 4). Across all eyes with available data at years 1, 2 and 3 postimplantation, 36.9% (*n* = 38/103), 42.0% (*n* = 34/81) and 48.3% (*n* = 29/60) of eyes that achieved overall success (i.e. sum of qualified and complete success, as defined in the *Outcomes* section) had undergone needling.

In the POAG subgroup at years 1, 2 and 3 postimplantation, 34.6% (*n* = 27/78), 37.1% (*n* = 23/62) and 39.0% (*n* = 16/41) of eyes that achieved overall success had undergone needling, respectively.

### Safety

Considering that anonymized data were collected retrospectively in this study, only AESIs, that is AEs identified during prior clinical studies of the gel stent, were documented. Of 212 eyes that underwent implantation of the gel stent, 15 (7.1%) reported intraoperative complications, with anterior chamber bleeding as the most common complication (*n* = 8/212; 3.8%).

Overall, 31/212 (14.6%) eyes experienced a total of 46 postoperative AEs (Table 5). Of the 46 AEs, 5 AEs noted in 2 eyes were classified as serious: shallow anterior chamber with iridocorneal touch, bleb leak, endophthalmitis, persistent numeric hypotony (defined as IOP <6 mmHg present at 2 consecutive postoperative visits >30 days apart) and clinical hypotony (defined as vision reduction of ≥2 lines related to macular changes consistent with hypotony maculopathy [macular folds], optic disc oedema and/or serious choroidal detachments because of IOP <6 mmHg).

**Table 5:** Intraoperative complications and postoperative AESIs.^a^

Intraoperative complications, *n* (%)^b^	Total (*N* = 212)	Primary eye (*N* = 174)
Anterior chamber bleeding	8 (3.8)	5 (2.9)
Subconjunctival bleeding obscuring view	1 (0.5)	0
**Postoperative AESIs, *n* (%)**		
Total number of eyes with any AEs	31 (14.6)^f^	27 (15.5)
Secondary surgical intervention	26 (12.3)	22 (12.6)
Trabeculectomy	13 (6.1)	10 (5.7)
Second gel stent	3 (1.4)	3 (1.7)
Selective laser trabeculoplasty	4 (1.9)	4 (2.3)
Argon laser trabeculoplasty	1 (0.5)	1 (0.6)
Ultrasound circular cyclocoagulation	2 (0.9)	0
Other unspecified procedures	3 (1.4)	3 (1.7)
Shallow anterior chamber with peripheral iridocorneal touch	3 (1.4)	3 (1.7)
Bleb leak	1 (0.5)	0
Corneal decompensation/oedema (<30 days)^c^	6 (2.8)	6 (3.4)
Dacryocystitis	1 (0.5)	1 (0.6)
Endophthalmitis	1 (0.5)	1 (0.6)
Hyphema	4 (1.9)	3 (1.7)
Hypotony, persistent^d^	1 (0.5)	1 (0.6)
Hypotony, clinical^e^	4 (1.9)	4 (2.3)
Implant exposure or extrusion	4 (1.9)	3 (1.7)
Implant fracture	2 (0.9)	2 (1.1)
Implant migration	2 (0.9)	2 (1.1)
Implant repositioning requiring surgical intervention	3 (1.4)	3 (1.7)
Implant touching iris	5 (2.4)	3 (1.7)
Macular oedema	1 (0.5)	1 (0.6)
Macular hole	1 (0.5)	1 (0.6)
Periorbital cellulitis	1 (0.5)	1 (0.6)
Ptosis	1 (0.5)	0
Retinal vein occlusion	1 (0.5)	1 (0.6)
Scleritis	1 (0.5)	0
Ulcerative keratitis	1 (0.5)	1 (0.6)
Vitreous haemorrhage	2 (0.9)	1 (0.6)

AE = adverse event, AESI = adverse event of special interest, phaco = phacoemulsification with intraocular lens placement.

^a^ Unless otherwise noted, an AESI was counted only once if occurred at more than one consecutive visit and the non‐serious/serious classification remained consistent. An AESI was considered as a separate event if it resolved and then returned (i.e. was reported at non‐consecutive visits).

^b^ Eight other complications were reported, but because of the anonymized nature of the study, it was not possible to ascertain their nature and categorize them.

^c^ Each event of corneal decompensation/oedema event or anterior chamber defect in which the subcategory changed was counted as a new event. No corneal AEs were reported beyond 30 days.

^d^ Defined as numeric IOP <6 mmHg, present at 2 consecutive postoperative visits >30 days apart.

^e^ Defined as vision reduction (≥2 lines) related to macular changes consistent with hypotony maculopathy (macular folds), optic disc oedema and/or serious choroidal detachments because of IOP <6 mmHg.

^f^ Two (0.9%) eyes had a total of 5 serious AEs (anterior chamber defects, bleb leak, endophthalmitis, persistent hypotony and clinical hypotony).

**Fig. 6:**
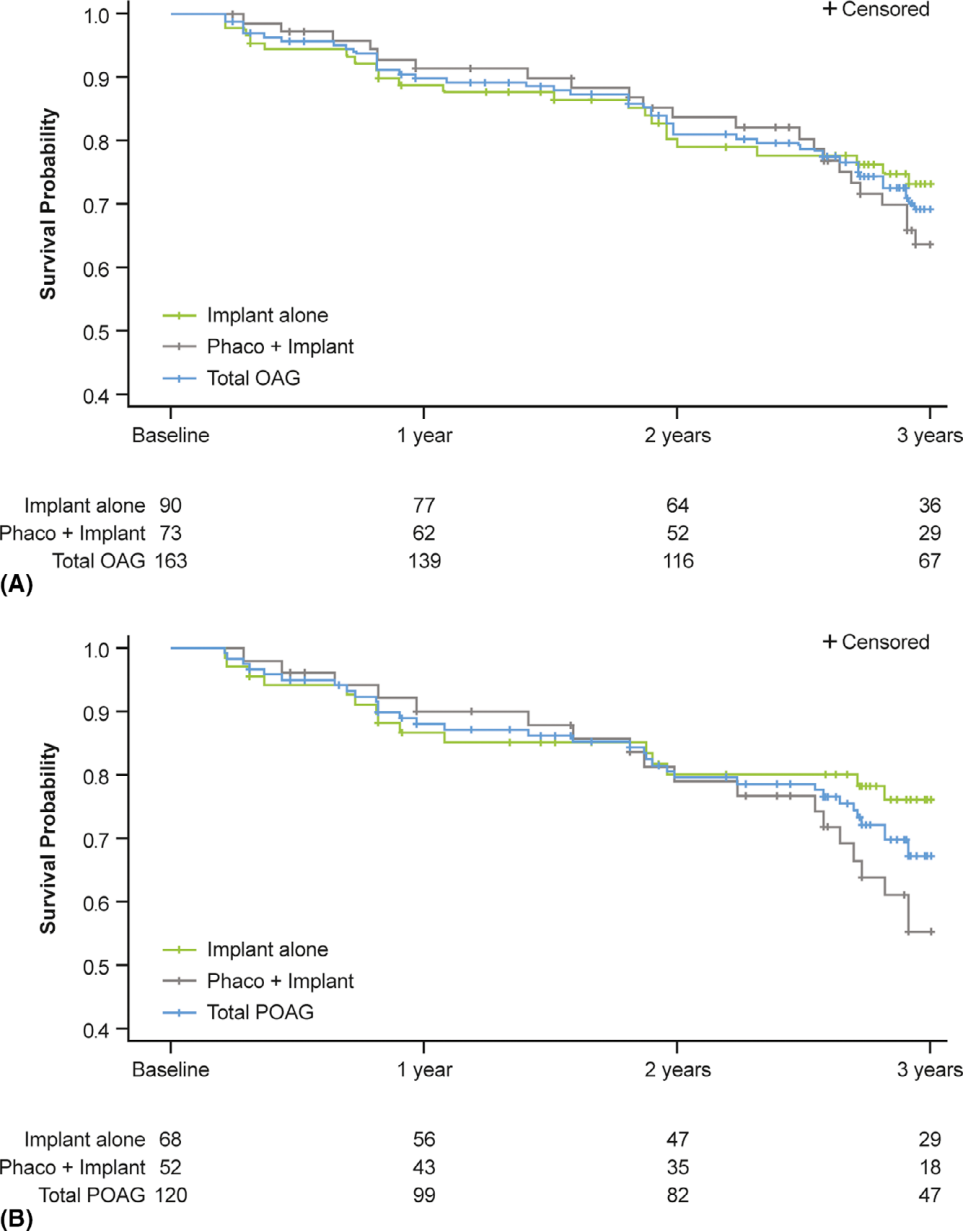
Kaplan–Meier curve showing the estimated probability of maintaining overall success at 3 years following gel stent implantation in the effectiveness^a^ OAG population with both IOP measurement and number of topical IOP‐lowering medications recorded at baseline (*n* = 163^b^) (A) and patient subgroup with POAG (*n* = 120) (B). Overall success was defined as a primary eye achieving complete success (≥20% IOP reduction from medicated baseline without SSI, clinical hypotony [as defined in the *Outcomes* section] or topical IOP‐lowering medications) or qualified success (≥20% IOP reduction from medicated baseline without SSI or clinical hypotony, while remaining on the same number or fewer topical IOP‐lowering medications), excluding eyes that required more IOP‐lowering medications postimplantation, compared with baseline. Failing to achieve the overall success at any time during postimplantation follow‐up was considered as failure. Primary eyes were considered as censored in the Kaplan–Meier analysis when their follow‐up period (including completing the study) ended before they failed. ^a^All effectiveness analyses included one eye per patient; in cases of bilateral implantation, only the first implanted eye was analysed. ^b^This analysis was based on the primary outcome measures and thus excluded 11 patients who did not have both IOP and IOP‐lowering medication count data at baseline. IOP = intraocular pressure, OAG = open‐angle glaucoma, phaco = phacoemulsification with intraocular lens placement, POAG = primary open‐angle glaucoma, SSI = glaucoma‐related secondary surgical intervention.

In total, 5 (2.4%), 2 (1.1%) and 4 (5.1%) eyes underwent open‐conjunctiva bleb revision once within the first, second and fourth year postimplantation, respectively; no open‐conjunctiva bleb revisions were reported during the third year. During the study period, 26 (12.3%) eyes required SSI, with trabeculectomy being the most common (*n* = 13) (Table 5). The mean (SD) time to SSI was 14.5 (11.3) months (Fig. 6). The survival probability in the OAG population and patient subgroup with POAG was ~70% and >65% at 3 years, and numerically higher in eyes that received the implant alone, compared with eyes that underwent phacoemulsification and gel stent implantation (Fig. 6).

As expected, mean (SD) BCVA improved from baseline to postimplantation at all time‐points in the phaco + implant subgroup. Although visual field assessment schedules were not uniform across sites, there were no statistically significant changes in mean deviation of the available visual fields from baseline to year 1 (p ≥ 0.0747), year 1 to year 2 (p ≥ 0.1021) and year 2 to year 3 (p ≥ 0.2527) in both treatment groups. Among patients with data available between 24 and 36 months (*n* = 77), the overall mean changes in visual field mean deviation were 0.4 (4.8) and −0.7 (3.6) dB in the implant alone and phaco + implant subgroups, from −10.0 (8.1) and −8.9 (7.0) dB at baseline, respectively.

## Discussion

Presented herein are findings of a retrospective multicentre study with 3‐year outcomes following gel stent implantation either as a standalone procedure or in combination with phacoemulsification. The European, primarily Caucasian cohort composed of 73.6% of patients with a diagnosis of POAG, which contributed 212 implanted eyes, was evaluated retrospectively. The mean (SD) IOP and the mean number of medications in the effectiveness population (163 eyes) decreased from 20.7 (5.1) mmHg and 2.5 (1.0) at baseline (*n* = 163) to 13.9 (4.3) mmHg (a 37.9% reduction) and 1.1 (1.2) at 3 years (primary outcome measures), respectively. We also reported sustained effectiveness at the 4‐year mark, albeit in a smaller cohort.

Three‐year results from our multicentre study appear to be similar to those of published studies reporting 2‐year (single‐ and multicentre studies) (Reitsamer et al. 2019; Gabbay et al. 2020) and 3‐year (single‐centre study) (Gillmann et al. 2020a) data. In a prospective, nonrandomized, single‐centre study (Gillman et al. 2020a) of 92 eyes with OAG (POAG, 40.2%) that received the implant alone (28.3%) or phaco + implant (71.3%), mean (SD) medicated IOP decreased by 37.0% from 20.8 (7.4) mmHg at baseline to 13.1 (3.4) mmHg at 3 years (p < 0.01), and the mean (SD) number of medications dropped from 1.9 (1.3) to 0.4 (0.9), respectively (p < 0.001). In the prospective, nonrandomized, multicentre APEX study (Reitsamer et al. 2019), 202 eyes with POAG received the implant alone (56.4%) or phaco + implant (43.6%); overall, mean (SD) medicated IOP was reduced from 21.4 (3.6) at baseline to 15.2 (4.2) mmHg at 2 years, with a mean per cent reduction of 27.8%, and the mean (SD) IOP‐lowering medication count decreased from 2.7 (0.9) at baseline to 1.1 (1.2), respectively (p < 0.001 for both). In a retrospective, noncomparative study of 151 eyes (primarily POAG, 84.1%) that received the gel implant alone (62.3%) or phaco + implant (37.7%) at a single centre (Gabbay et al. 2020), a 34.6% reduction in mean (SD) IOP from a baseline of 22.1 (6.5) mmHg to 14.5 (3.3) mmHg was reported at 2 years, along with a reduction in the mean number of IOP‐lowering medications from 2.8 (1.1) to 0.5 (1.0) (p < 0.001 for both).

In the present study, outcomes at 3 years were generally similar in the subset of eyes with POAG and the total population and comparable with those observed at 2 years in the aforementioned studies (Mansouri et al. 2019; Reitsamer et al. 2019; Gabbay et al. 2020). The scatter plots further showed that of all eyes with data available at 3 years postimplantation, only 2 had postoperative IOPs above 21 mmHg. These 2 outliers were not present in the POAG subgroup analysis, indicating that they had secondary glaucomas. The one unusual finding we report is the higher number of eyes with complete success, compared with those achieving qualified success; we were unable to find any correlation to other factors such as needling to explain this finding.

Although a formal statistical comparison of the treatment subgroups was not performed, the gel stent effectively reduced IOP and the IOP‐lowering medication count at 3 years, whether implanted alone or in combination with cataract surgery. Nonetheless, the phaco + implant subgroup had a lower complete success rate (20.0%), compared with the qualified success rate (40.0%), suggesting that more medications were needed to achieve success.

Consistent with previous reports, our study also indicates that needling may be required in a proportion of eyes in order to achieve adequate IOP lowering. Needling is a postoperative management procedure that should be undertaken as necessary to improve aqueous flow into the bleb and lower IOP, per the American Academy of Ophthalmology in their Preferred Practice Patterns for POAG (American Academy of Ophthalmology 2020). Of note, late needlings (after >2 years) were as successful as early needlings (<1 year). By year 3 postimplantation, 43% of eyes had been needled (most occurring within the first 12 months), in line with needling rates of 37.7%, 41.1% and 45.0% reported at 2 years by Gabbay et al. (2020), Reitsamer et al. (2019) and Mansouri et al. (2019), respectively. The mean number of needling per eye observed herein (1.3) was also similar to that reported by Reitsamer et al. (1.6) (Reitsamer et al. 2019) or estimated based on data from Mansouri et al (1.6) (Mansouri et al. 2019). Bleb revision may also be an option to rehabilitate failing blebs. The proportion of eyes requiring bleb revision reported herein (5.2% overall during the 4‐year study) was in line with that of Gabbay et al. (4.6%) (Gabbay et al. 2020).

Overall, the intra‐ and postoperative AESIs recorded through 48 months were consistent with product information and those previously reported in the literature (Allergan 2016; Allergan 2017; Mansouri et al. 2019; Reitsamer et al. 2019; Gabbay et al. 2020). Moreover, the proportion of eyes requiring SSI (12.3%) at 3 years was comparable with that reported by Mansouri et al. (11.4%) at 2 years (Mansouri et al. 2019), higher than that reported by Reitsamer et al. (6.4%) at 2 years (Reitsamer et al. 2019), but lower than that reported by Gabbay et al. (25%) at 2 years (Gabbay et al. 2020).

Our study had some limitations: being a retrospective chart review, the availability of data was variable across sites, based on differences in follow‐up regimens from country to country. Additionally, the anonymized nature of data collection prevented querying of data once it was entered into the study database, per the European Regulation (EU 2016/679) on protection of personal data, affecting the ability to sub‐analyse by site (e.g. needling rate). In addition, the findings reflect results of surgical approaches that may not represent current preoperative preparation methods, refinements in surgical techniques, and peri‐ and postoperative practices. The gel stent‐related surgical techniques are indeed evolving rapidly, with a major emphasis on the implant’s distal end being placed supra‐ or sub‐Tenon, which can be achieved via ab‐interno or ab‐externo approaches, with or without conjunctival dissection (Panarelli et al. 2020; Vera et al. 2020). Other practices, such as preoperative control of inflammation by avoiding topicals with preservatives and/or using steroids, primary needling at the time of implantation and modulation of fibrosis with a 5‐fluorouracil regimen, are being incorporated into clinical practice (Lenzhofer et al. 2019; Vera et al. 2019; Panarelli et al. 2020; Vera et al. 2020; Wałek et al. 2020). Future studies will allow for a better understanding of the impact of these different approaches and practices on outcomes.

In summary, our results suggest that the traditional method of ab‐interno placement of the gel stent without conjunctival dissection, used in the vast majority (96.7%) of eyes herein and in use for over a decade (Schlenker et al. 2017), provides consistent effectiveness in reducing IOP and the number of IOP‐lowering medications over 3 years, with a predictable and acceptable safety profile.
